# Age‐ and gender‐specific disease distribution and the diagnostic accuracy of CT for resected anterior mediastinal lesions

**DOI:** 10.1111/1759-7714.13081

**Published:** 2019-04-29

**Authors:** Ju Gang Nam, Jin Mo Goo, Chang Min Park, Hyun‐Ju Lee, Chang Hyun Lee, Soon Ho Yoon

**Affiliations:** ^1^ Radiology Seoul National University Hospital Seoul Korea; ^2^ College of Medicine Seoul National University Seoul Korea; ^3^ Institute of Radiation Medicine Seoul National University Medical Research Center Seoul Korea

**Keywords:** Anterior mediastinal neoplasms, multidetector computed tomography, thymic cyst, thymic carcinoma, thymoma

## Abstract

**Background:**

Anterior mediastinal lesions account for approximately half of all mediastinal masses and computed tomography (CT) is known to exhibit limited differentiating performance. Our aim was to evaluate the age‐ and gender‐specific distribution of anterior mediastinal lesions and the diagnostic accuracy of multi‐detector CT (MDCT).

**Methods:**

This retrospective study included 549 consecutive patients with proven anterior mediastinal lesions and diagnostic MDCT scans. The age‐ and gender‐specific distribution of proven diagnoses and diagnostic accuracy were reviewed. The CT features of malignant and benign diseases having the lowest accuracy were compared with those of the most commonly misdiagnosed diseases.

**Results:**

The proportion of malignancy showed a V‐shape relationship with age (lowest, 52.7% [50s]). The most prevalent malignancies were lymphoma (20s), lymphoma/thymoma (30s), thymoma (40s–50s), and thymoma/thymic carcinoma (≥60s). The most prevalent benign diseases were thymic remnant/hyperplasia (20s–30s), and thymic bed cyst (≥40s). The first‐choice diagnostic accuracy of MDCT decreased with age regardless of gender: 75.4% (20s), 75.0% (30s), 67.8% (40s), 58.5% (50s), and 53.4% (≥60s), primarily due to incorrect diagnoses of thymic bed cyst and thymic carcinoma (accuracy, 42.3% and 30.5%), which were prevalent in older patients and mostly misdiagnosed as thymoma. The most powerful differentiating MDCT features were water attenuation (≤20 HU) (OR, 42.7 [95%CI, 8.8–‐208.3], *P* < 0.001) for thymic bed cyst and mediastinal lymphadenopathy (6.8 [1.7–27.2], *P* = 0.006) for thymic carcinoma, but both showed low sensitivity (34.5% and 18.6%, respectively).

**Conclusions:**

MDCT accuracy depended on age, owing to the age‐specific distribution of thymic carcinoma and thymic bed cyst, which frequently lacks distinguishable CT features from thymoma.

## Introduction

Anterior mediastinal lesions account for approximately half of all mediastinal masses,^1^, ^2^ which include various disease entities from benign cysts to cancers.^3^, ^4^ Computed‐tomography (CT) has been conventionally used for primary evaluation of anterior mediastinal lesions, but is known to exhibit limited differentiating performance. The previous study reported that single‐channel chest CT offers modest diagnostic accuracy, with a correct first‐choice diagnosis of 61% (95% CI, 52–69%; 78/126).^2^ As a result, some anterior mediastinal pathologies still receive incorrect imaging diagnoses, leading to nontherapeutic thymectomy in 22% to 44% of cases.^5^, ^6^

The distribution of anterior mediastinal lesions depends on age and gender^3^ and chest CT scan is primarily obtained on multi‐detector CT (MDCT) scanners in current routine practice. Nevertheless, how the diagnostic performance of chest MDCT for anterior mediastinal lesions is affected by age and gender in a sufficient number of cases has been limitedly evaluated. Thus, the purpose of this study was to evaluate the age‐ and sex‐specific distribution of anterior mediastinal lesions and MDCT diagnostic accuracy.

## Methods

### Patients

**Figure 1:**
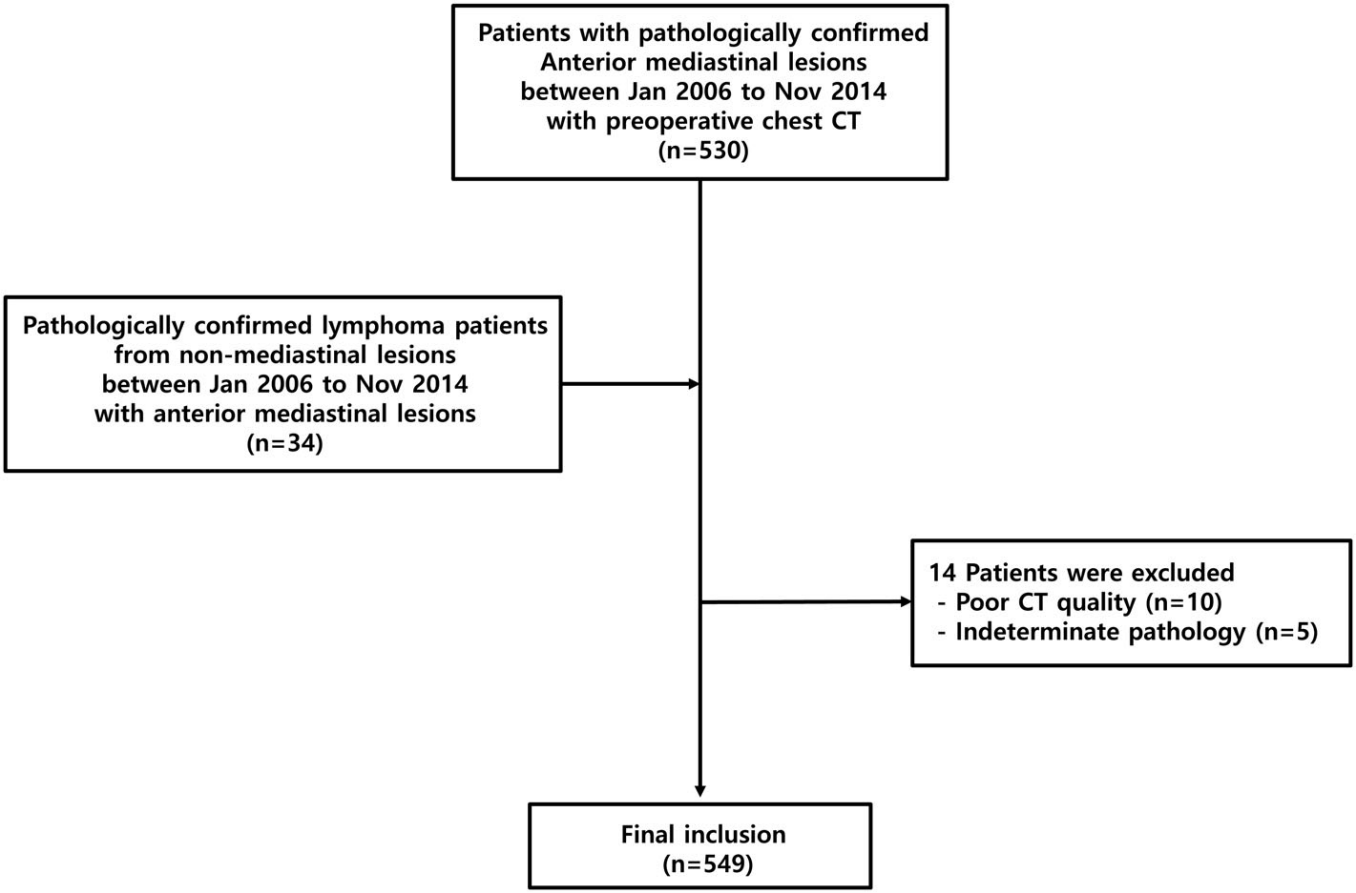
Flowchart for the inclusion of our study population.

The Institutional Review Board of our hospital approved this retrospective study and the requirement for informed consent was waived. According to a pathologic database search performed by the study coordinator (J.G.N.), patients who met the following inclusion criteria were included (Fig 1): (i) pathologically proven anterior mediastinal lesion between January 2006 and November 2014; (ii) MDCT images obtained with the standardized protocol for chest CT; (iii) a mean interval between pathologic diagnosis and MDCT of no longer than 1.5 months; and (iv) no history of previous treatment such as chemotherapy. For some lymphoma patients (34/78), a pathologic confirmation was obtained from a body part other than the anterior mediastinum, and all were cervical lymph nodes. Patients with poor CT image quality and indeterminate pathologic diagnosis were excluded. Finally, 549 consecutive patients with a mean age (± SD) of 50.6 ± 14.9 years (278 men [mean age, 51.2 ± 15.3 years; age range, 17–84 years] and 271 women [50.0 ± 14.5; 16–77]) were included.

### MDCT image acquisition

Because of the rapid development of MDCT technology, CT scans were performed using various MDCT scanners with 4‐(*n* = 63), 8‐(*n* = 79), 16‐(*n* = 96), 64‐(*n* = 282), and 320‐rows (*n* = 29). The scanning parameters were a tube voltage of 120 kVp with automatic exposure control of the tube current, slice thickness of 1.0–3.0 mm, and a reconstruction interval of 0.6–3.0 mm in most of CT scans, along with 3 mm coronal multi‐planar reformatted images. Patients were scanned craniocaudally from the lung apex to the costophrenic angle in the supine position at full inspiration during a single breath‐hold. A total of 90 mL of iodinated contrast media was injected into an antecubital vein at 3.0 mL/second, followed by a saline chase of 30 mL at the same rate. The postcontrast scan was usually taken 60 seconds after the contrast media injection.

### Analysis of MDCT diagnostic accuracy

During the study period, one of four expert thoracic radiologists (J.M.G., C.H.L., H.J.L., and C.M.P., with 12–26 years of clinical experience) primarily read chest CT scans and wrote the formal radiology reports in routine clinical practice. One author (J.G.N.) extracted the first‐choice and top three differential diagnoses from the formal reports and assessed the proportion of cases where the radiologic diagnosis was consistent with the pathologic diagnosis. We excluded a total of 17 cases where the radiology report was made after pathologic confirmation.

Histologically‐proven disease entities of anterior mediastinal lesions were classified as malignant or benign. The malignant diseases comprised aggressive lymphoma (*n* = 66), low‐grade mucosa‐associated lymphoid tissue lymphoma (*n* = 12), malignant germ cell tumor (*n* = 9), thymoma (*n* = 182), thymic carcinoma (*n* = 59), lung cancer (*n* = 10), and other rare tumors including angiosarcoma (*n* = 2), metastasis (n = 4), neuroendocrine tumor (*n* = 5), mesothelioma (*n* = 1), and other sarcoma (*n* = 2). The benign diseases comprised thymic or bronchogenic cyst (*n* = 142), thymic hyperplasia or remnant (*n* = 15), benign teratoma (*n* = 30), and other rare benign diseases, including lymphangioma (*n* = 2), thymolipoma (*n* = 2), perivascular epithelioid cell tumor (*n* = 1), Castleman disease (*n* = 1), inflammatory lesion (*n* = 3), and parathyroid cyst (*n* = 1). Thymic cysts and bronchogenic cysts were grouped together as thymic bed cysts. The proportions of diseases were assessed according to age in 10‐year intervals and gender.

### Analysis of MDCT findings

All MDCT images were retrospectively reviewed by two radiologists (J.G.N. and S.H.Y., with three and 12 years of experience in the interpretation of thoracic imaging) in consensus. CT images were evaluated for morphologic features, ancillary findings, and enhancement features on a picture archiving and communication system workstation (Infinitt, Seoul, Korea) in soft‐tissue window settings (width, 400 HU; level, 20 HU). The location of tumors was categorized as superior or inferior and as median or eccentric according to the relative location of the tumor epicenter and the heart.^7^ The lesion was defined to have a superior location if the epicenter of the lesion was located above the heart and to have an eccentric location when the epicenter was located off‐midline more than a half‐width of the lesion. The size of the tumor was measured bidirectionally and the contour, margin, and shape of the tumor were evaluated. The shape was classified as round if the long‐to‐short‐axis ratio was <1.5, and oval or irregular if the dimension ratio was ≥1.5.^2^, ^7^, ^8^ The presence of a focal cystic or necrotic portion, calcification, gross fat, or adjacent organ invasion was also analyzed.^2^, ^7^, ^8^ Adjacent organ invasion included invasion of the heart, lung, major vessels, or chest wall.^7^, ^8^ The ancillary findings included the presence of pleural effusion, pericardial effusion, mediastinal or hilar lymph node (LN) enlargement (short diameter ≥ 10 mm), distant metastasis, or satellite lesions.^2^, ^8^

The CT attenuation value of the lesion was measured using a circular region‐of‐interest in precontrast (*n* = 406) and postcontrast (*n* = 481) axial images. If a postcontrast‐enhanced scan was performed, the attenuation value was obtained from the area of the lesion that had the greatest attenuation on the postcontrast‐enhanced images, excluding the necrotic or cystic portion. Based on the previous cut off,^9^ the proportion of lesions with water attenuation of 20 HU or less in pre‐ and postcontrast images was calculated.

### Statistical analysis

The proportions of disease entities according to age and gender were compared using the chi‐square test. The chi‐square test and Fisher exact test were used for examining statistical differences between the proportion of correct first‐choice diagnoses of a particular disease and that of malignant/benign diseases other than that disease. MDCT features were compared between thymic bed cyst and thymoma, and between thymoma and thymic carcinoma, using the Student *t*‐test or the chi‐square test, as appropriate. Variables with a *P*‐value <0.10 in the univariate analysis were included in the multivariate logistic regression analysis through the enter method. To examine the impact of differentiating CT features on the MDCT diagnosis, the proportion of correct diagnoses was compared using the Fisher exact test according to the presence of the significant CT features. Statistical analyses were performed using MedCalc version 15.8 (MedCalc, Mariakerke, Belgium). For all tests, *P‐*values <0.05 were considered statistically significant.

## RESULTS

### Age‐ and gender‐specific distribution of diseases

**Figure 2:**
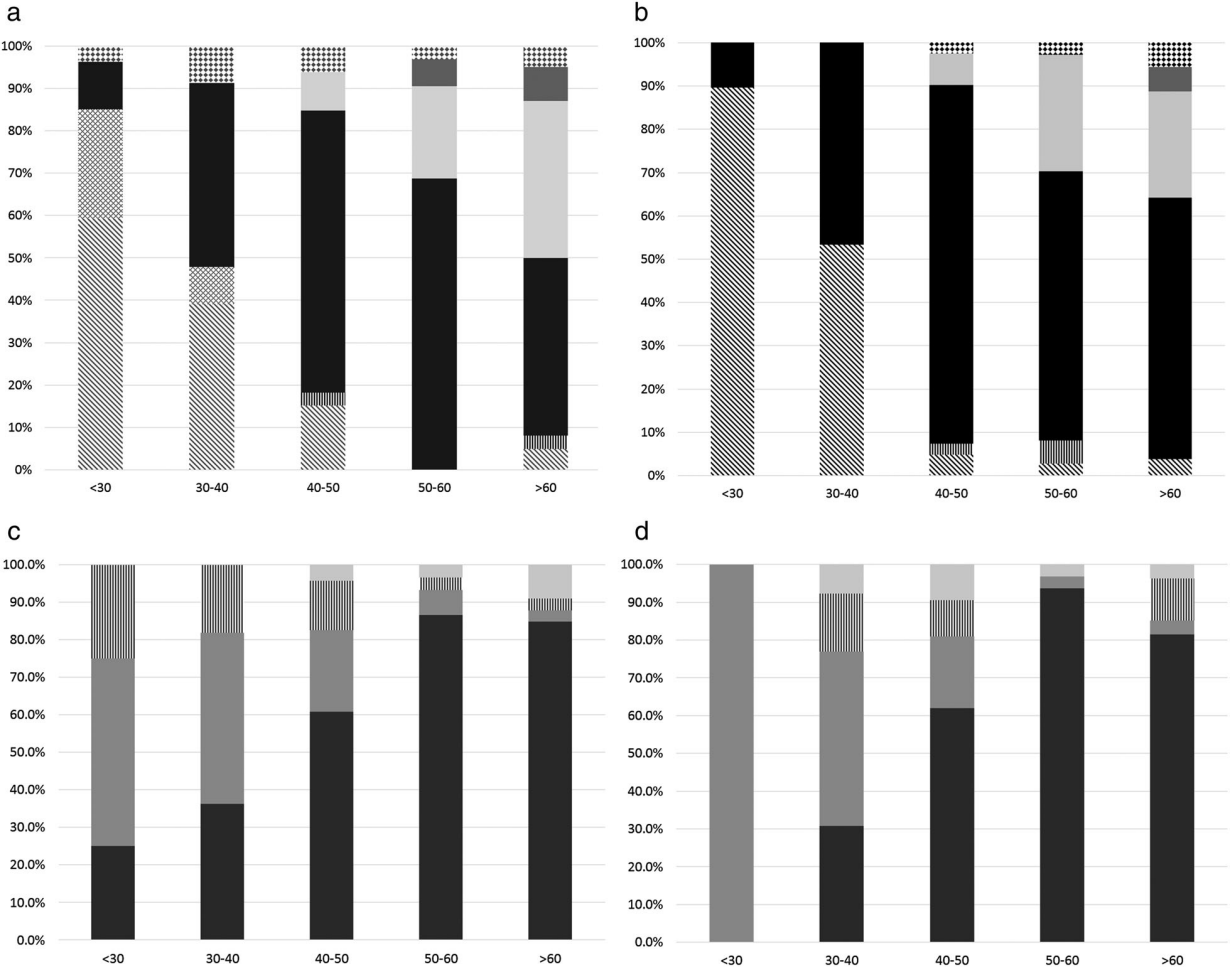
The age‐specific distribution of malignant (**a** and **b** for male and female patients, respectively) and benign (**c** and **d**) anterior mediastinal pathologies. (**a**, **b**) (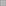) others (malignant); (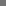) lung cancer; (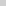) thymic carcinoma; (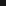) thymoma; (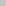) malignant germ cell tumor; (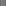) low grade MALtoma; (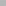) aggressive lymphoma. (**c**, **d**) (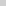) others (benign); (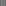) benign teratoma; (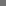) thymic hyperplasia or remnant; (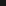) thymic bed cyst.

The comprehensive age‐ and gender‐specific distribution is shown in Figure 2 and Table S1. Malignancy showed a V‐shape relationship with age: the proportion of malignancy were 88.9% (56/63), 61.3% (38/62), 62.7% (74/118), 52.7% (69/131), and 65.7% (115/175) for patients <30, in their 30s, 40s, 50s, and 60s or older, respectively.

Among patients under 30 years, aggressive lymphoma was the most prevalent malignancy for both males and females (59.3% [16/27] and 89.7% [26/29], respectively), followed by malignant germ cell tumor (25.9%, 7/27) in males and thymoma in females (10.3%, 3/29) with the exclusive presence of malignant germ cell tumor in male patients under 30 years (*P* = 0.012). As age increased towards the 40s, the proportions of lymphoma decreased, while thymoma increased to account for the major proportion of malignant anterior mediastinal lesions. Furthermore, the portion of thymic carcinoma tended to gradually increase in malignancies as age increased.

The most prevalent benign diseases were thymic hyperplasia or remnant in their 20s–30s and thymic bed cyst in their 40s. Thymic bed cyst is predominant pathology of benign anterior mediastinal lesions in 50s or older, irrespective of gender.

### MDCT diagnostic accuracy

**Figure 3:**
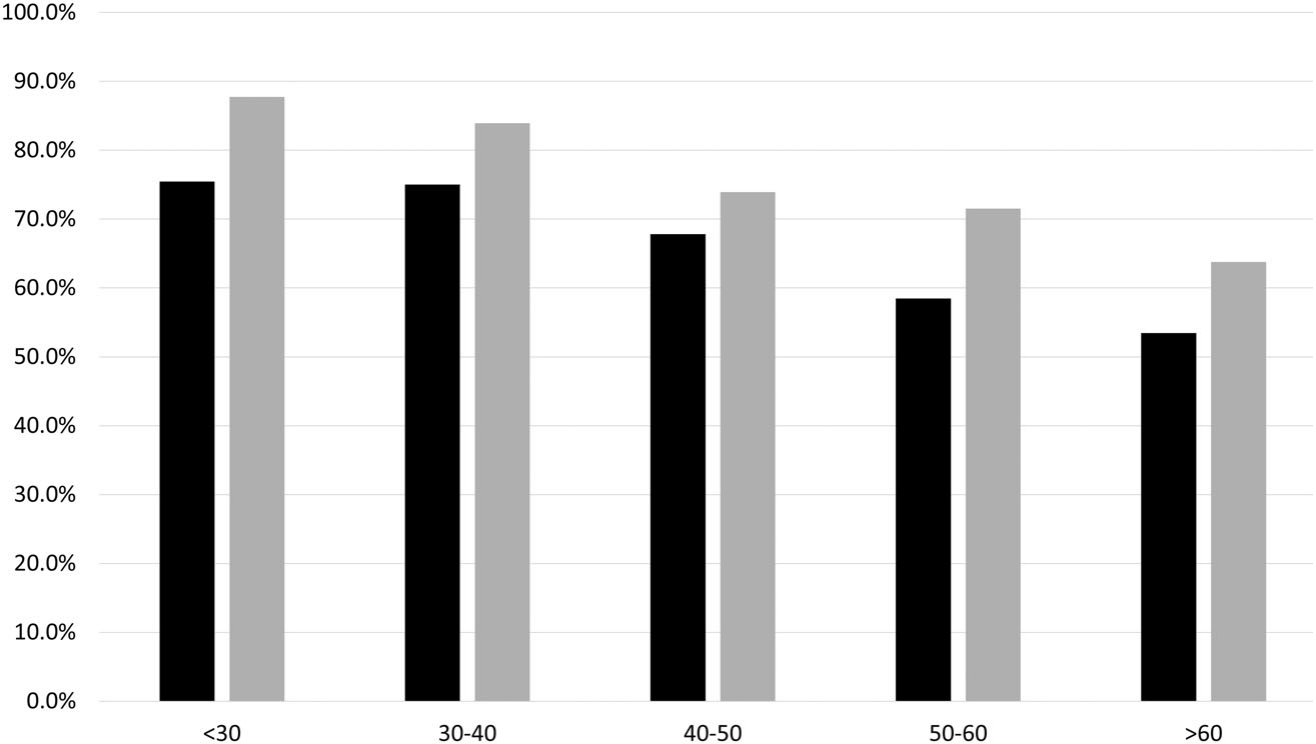
The age‐ and gender‐dependent MDCT accuracy for anterior mediastinal pathologies. (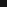) First‐choice differential diagnosis and (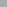) Top three differential diagnoses.

The overall proportions of correct first‐choice and top three differential diagnoses were 62.4% (332/532, 95% CI, 58.1–66.3%) and 72.6% (386/532, 95% CI, 68.5–76.1%), respectively (Table 1). The proportion of correct first‐choice diagnoses decreased as age increased (75.4% [43/57], 75.0% [42/56], 67.8% [78/115], 58.5% [76/130], and 53.4% [93/174] for patients <30, in their 30s, 40s, 50s, and ≥60s, respectively) (Table 2, Fig 3). The proportion of correct first‐choice diagnoses was significantly higher for malignancies (70.7%, 237/335) than for benign disease (48.2%, 95/197; *P* = 0.0001).

**Table 1:** Disease‐specific diagnostic accuracy of preoperative MDCT in predicting pathologically confirmed anterior mediastinal lesions

		Diagnostic accuracy		
	Patients No.	First‐choice differential diagnosis	Top three differential diagnoses	*P‐* value†
Malignancy	335	70.7% (237/335)	79.4% (266/335)	
Lymphoma‡	61	65.6% (40/61)	77.0% (47/61)	0.409
Malignant germ cell tumor	9	66.7% (6/9)	100% (9/9)	0.921
Thymoma	182	90.1% (164/182)	95.1% (173/182)	<0.0001
Thymic carcinoma	59	30.5% (18/59)	44.1% (26/59)	<0.0001
Lung cancer	10	80.0% (8/10)	90.0% (9/10)	0.764
Other rare malignancies	14	7.1% (1/14)	14.3% (2/14)	<0.0001
Benign disease	197	48.2% (95/197)	60.9% (120/197)	
Thymic bed cyst	142	42.3% (60/142)	57.7% (82/142)	0.011
Thymic hyperplasia or remnant	15	53.3% (8/15)	60.0% (9/15)	0.886
Benign teratoma	30	83.7% (25/30)	86.7% (26/30)	0.0001
Other rare benign diseases	10	20.0% (2/10)	30.0% (3/10)	0.131
Total	532	62.4% (332/532)	72.6% (386/532)	

† *P*‐values are for the significances of the differences between the diagnostic accuracy of the corresponding disease compared with other malignant/benign diseases excluding each disease; evaluated from the χ^2^ test or Fisher's exact test, as appropriate. Significant *P*‐values (<0.05) were underlined.

‡ For lymphoma patients, patients with pathologic confirmation of the disease ahead of chest MDCT evaluation were excluded (17 out of 78).

**Table 2:** Age‐ and gender‐specific diagnostic accuracy of preoperative MDCT

	First‐choice differential diagnosis				Top three differential diagnoses			
Age	Total	Male	Female	*P‐*value†	Total	Male	Female	*P*‐value
<30	75.4% (43/57)	75.9% (22/29)	75.0% (21/28)	0.816	87.7% (50/57)	93.1% (27/29)	82.1% (23/28)	0.392
30–40	75.0% (42/56)	77.4% (24/31)	72.0% (18/25)	0.877	83.9% (47/56)	83.9% (26/31)	84.0% (21/25)	0.724
40–50	67.8% (78/115)	63.6% (35/55)	71.7% (43/60)	0.471	73.9% (85/115)	69.1% (38/55)	78.3% (47/60)	0.360
50–60	58.5% (76/130)	56.5% (35/62)	60.3% (41/68)	0.790	71.5% (93/130)	69.4% (43/62)	73.5% (50/68)	0.740
>60	53.4% (93/174)	50.0% (47/94)	57.5% (46/80)	0.403	63.8% (111/174)	60.6% (57/94)	67.5% (54/80)	0.435

† *P*‐values are for the significances of the differences between the diagnostic accuracy of the corresponding disease compared with the other gender; evaluated from the χ^2^ test or Fisher's exact test, as appropriate.

**Figure 4:**
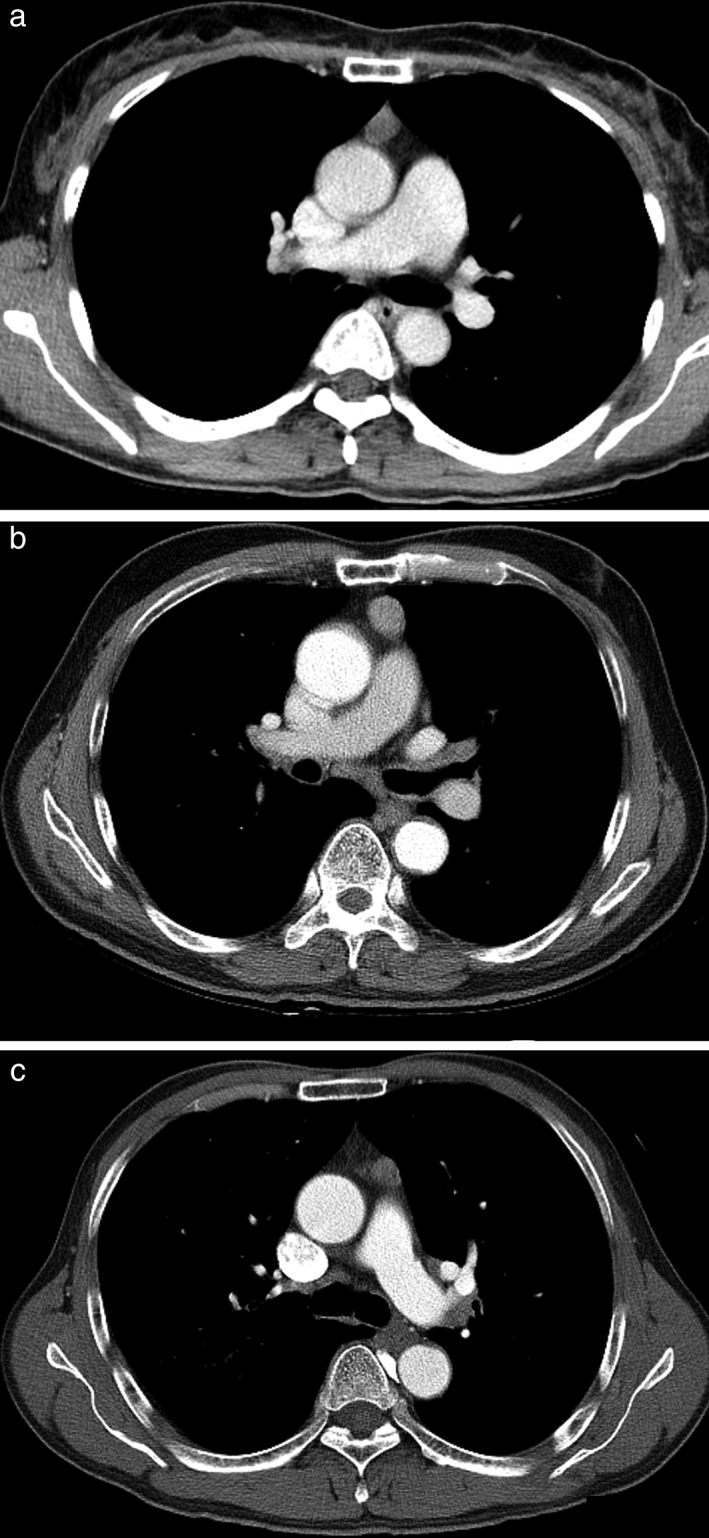
Three CT scans from three different patients showing the limitation of conventional CT in differentiating three pathologies. All scans showed 1 to 2 cm sized smooth‐contoured, median‐located, and soft tissue‐attenuated (>20 HU) anterior mediastinal lesions. The pathology indicated these to be thymic cyst (**a**), thymoma (**b**), and thymic carcinoma (**c**), respectively.

The age‐specific trend was mainly attributed to limitations in the MDCT diagnosis for thymic bed cyst and thymic carcinoma (Tables 1 and 2). The proportion of correct first‐choice diagnoses was significantly lower for thymic carcinoma (30.5%; 18/59) than for other malignant diseases (83.2%; 218/262, *P* < 0.0001), and for thymic bed cyst (42.3%; 60/142) than other benign diseases (73.3%; 33/45, *P* < 0.0001). Among the misinterpreted cases of those two diseases, the most common first differential diagnosis was thymoma (thymic bed cyst, 80.5%, 66/82; thymic carcinoma, 87.8%; 36/41) (Fig 4).

### CT features differentiating thymic bed cyst and thymic carcinoma from thymoma

*Thymic bed cyst versus thymoma*: Univariate analysis revealed that thymic bed cysts were more superiorly (*P* = 0.005) and medially located (*P* < 0.0001), smaller (*P* < 0.0001), more smoothly contoured (*P* < 0.0001), more well‐demarcated (*P* < 0.0001), more round‐shaped (*P* = 0.014), and water‐attenuated on pre‐ and postcontrast scans (HU ≤ 20) (*P* < 0.0001). On the other hand, thymomas showed more frequent focal cystic/necrotic changes (*P* < 0.0001), calcification (*P* < 0.0001), adjacent organ invasion (*P* = 0.0002), pleural effusion (*P* = 0.029), mediastinal LN enlargement (*P* = 0.046), or distant metastasis (*P* = 0.002) (Table 3).

**Table 3:** MDCT image features for difficult‐to‐diagnosis pathologies of anterior mediastinal lesions

				*P* value†	
	Thymic bed cyst	Thymoma	Thymic carcinoma	TC *versus* Tm	Tm *versus* TCa
Age >60	32.4% (46/142)	29.1% (53/182)	55.9% (33/59)	0.608	0.0003
Location					
Longitudinal					
Superior	91.5% (130/142)	80.2% (146/182)	89.8% (53/59)	0.005	0.092
Inferior	8.5% (12/142)	19.8% (36/182)	10.2% (6/59)		
Laterality					
Median location	104/142 (73.2%)	58/182 (31.9%)	39.0% (23/59)	<0.0001	0.487
Lesion characteristics					
Size (cm, mean ± SD)	2.86 ± 4.62	4.88 ± 2.10	5.00 ± 2.29	<0.0001	0.717
Contour					
Smooth	83.8% (119/142)	28.0% (51/182)	23.7% (14/59)	<0.0001	0.484
Lobulated	16.2% (23/142)	72.0% (131/182)	76.3% (45/59)		
Margin					
Well‐demarcated	89.4% (127/142)	46.7% (85/182)	25.4% (15/59)	<0.0001	<0.0001
Focal‐bulging	4.2% (6/142)	31.9% (58/182)	22.0% (13/59)		
Ill‐defined	6.3% (9/142)	21.4% (39/182)	52.5% (31/59)		
Shape					
Round	31.7% (45/142)	19.8% (36/182)	20.3% (12/59)	0.014	0.926
Oval/irregular	68.3% (97/142)	80.2% (146/182)	79.7% (47/59)		
Focal cystic/necrotic change	0.7% (1/142)	33.0% (60/182)	50.8% (30/59)	<0.0001	0.002
Calcification	2.8% (4/142)	17.6% (32/182)	13.6% (8/59)	<0.0001	0.519
Gross fat	0.0% (0/142)	0/0% (0/182)	0/0% (0/59)	—	
Adjacent organ invasion	0.0% (0/142)	10.3% (17/182)	33.9% (20/59)	0.0002	<0.0001
Ancillary features					
Pleural effusion	0.0% (0/142)	3.3% (6/182)	6.8% (4/55)	0.029	0.004
Pericardial effusion	0.0% (0/142)	0.5% (1/182)	8.5% (5/54)	0.378	0.098
Mediastinal LN enlargement	0.0% (0/142)	2.7% (5/182)	18.6% (11/59)	0.046	<0.0001
Distant metastasis	0.0% (0/142)	7.7% (14/182)	16.9% (10/59)	0.002	0.063
Satellite lesions	2.1% (3/142)	3.8% (7/182)	1.7% (1/59)	0.367	0.065
Enhancement pattern					
Attenuation (precontrast) (HU, mean ± SD)	34.3 ± 18.7	45.8 ± 11.6	45.7 ± 18.8	<0.0001	0.978
Water‐attenuated on precontrast scan‡	26.2% (28/107)	2.2% (3/138)	2.2% (1/46)	<0.0001	0.574
Attenuation (postcontrast) (HU, mean ± SD)	36.7 ± 20.4	86.8 ± 25.5	79.2 ± 22.2	<0.0001	0.049
Water‐attenuated on postcontrast scan†	23.8% (30/126)	0.0% (0/176)	0.0% (0/59)	<0.0001	—
Homogeneous enhancement	95.9% (118/123)	59.0% (102/173)	45.8% (27/54)	<0.0001	0.247

† *P*‐values are from the Student's *t*‐test or chi‐squared test, as appropriate. Significant *P*‐values (<0.05) were underlined.

‡ “Water‐attenuated” indicates with a CT attenuation not greater than 20 HU.

TC, thymic bed cyst; TCa, thymic carcinoma; Tm, thymoma.

**Figure 5:**
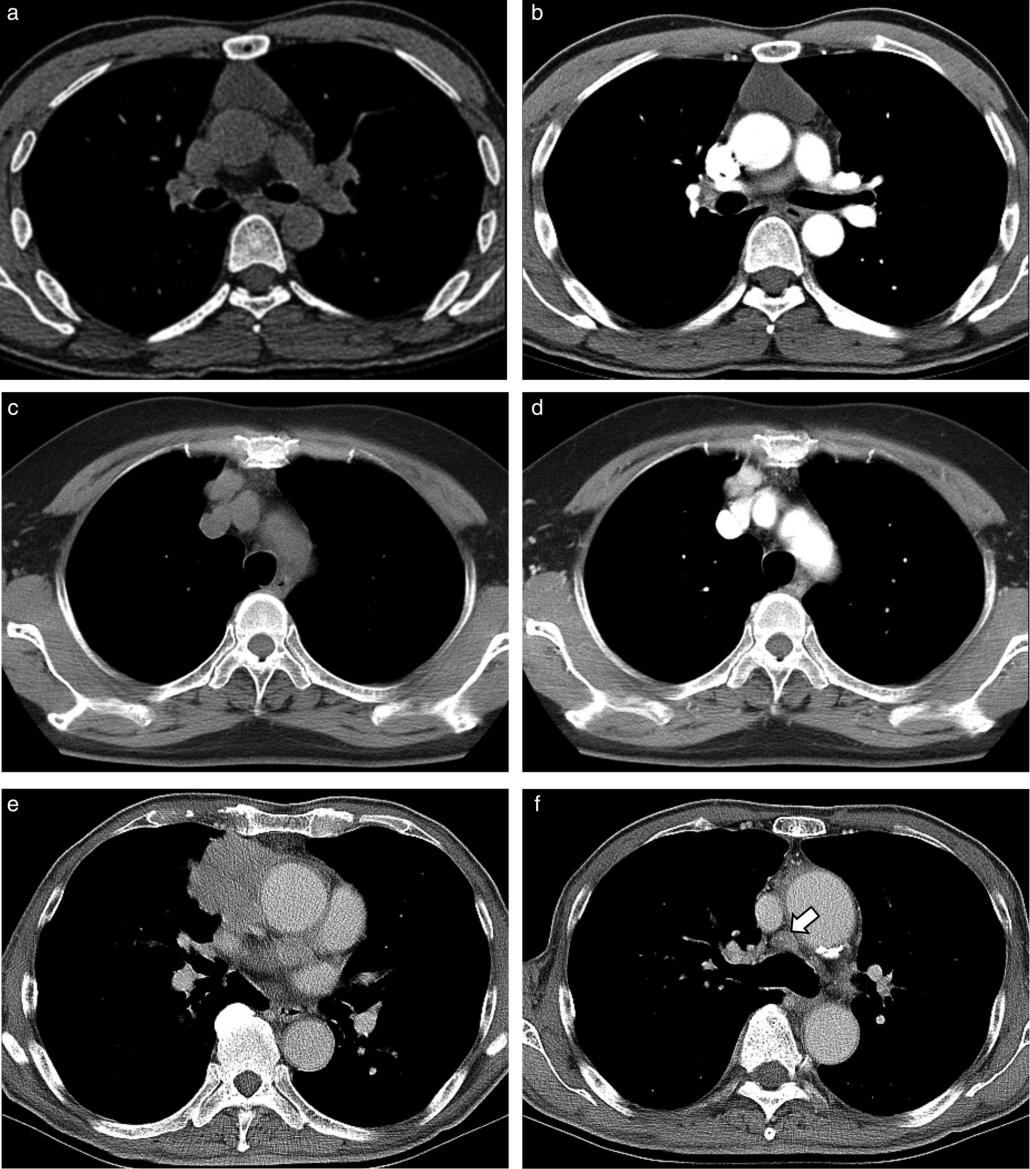
Three representative cases CT scans of three pathologies. (**a**, **b**) A 43‐year‐old male patient presented with a 4.3 cm sized unilocular cystic lesion confirmed to be a thymic cyst. The lesion showed smooth contour and well‐demarcated margin without any focal necrotic area, and was located in the median position. The attenuation of the lesion was less than 20 in both pre‐ and postcontrast scans. (**c**, **d**) A 67‐year‐old female patient presented with a 2.2 cm sized eccentric‐located lobulated‐contoured lesion without evidence of local invasion or distant metastasis. The lesion showed soft tissue‐attenuation on the precontrast scan (42 HU) and contrast enhancement (151 HU after contrast media injection). The lesion was confirmed to be thymoma after resection. (**e**, **f**) A 70‐year‐old male patient showed 10.9 cm sized mass with adjacent lung/venous invasion and mediastinal lymph node enlargement (arrowed). This lesion was surgically confirmed to be thymic carcinoma.

In the multivariate analysis (Table 4), water attenuation on (HU ≤ 20 on pre‐ or postcontrast CT) (OR, 42.74; 95% CI, 8.75–208.33; *P* < 0.001), a smooth lesion contour (OR, 7.35; 95% CI, 3.48–15.56; *P* < 0.001), no focal cystic/necrotic changes (OR, 5.08; 95% CI, 1.54–21.79; *P* = 0.009), a median location (OR, 2.42; 95% CI, 1.51–3.90; *P* < 0.001), and well‐defined margin (OR, 2.06; 95% CI, 1.18–3.60; *P* = 0.001) remained significantly associated with thymic bed cyst. The corresponding sensitivity and specificity of the most strongly differentiating CT feature, water attenuation, were 34.5% and 98.4%, respectively. Typical cases are presented in Figure 5.

**Table 4:** Multivariate analysis results of significant variables with the sensitivity and specificity to differentiate thymic bed cyst from thymoma and thymic carcinoma from thymoma

	Multivariate analysis results			Diagnostic performance	
Imaging findings	*P‐* value†	Odds ratio	95% CI	Sensitivity	Specificity
Differentiating thymic bed cyst from thymoma					
Water‐attenuated‡	<0.001	42.74	8.75–208.33	34.5% (49/142)	98.4% (179/182)
Smooth lesion contour	<0.001	7.35	3.48–15.56	83.8% (119/142)	72.0% (131/182)
Without focal cystic/necrotic change	0.009	5.8	1.54–21.79	99.3% (141/142)	33.0% (60/182)
Median location	<0.001	2.42	1.51–3.90	73.2% (104/142)	68.1% (124/182)
Well‐demarcated margin	0.011	2.06	1.18–3.60	89.4% (127/142)	53.2% (97/182)
Differentiating thymic carcinoma from thymoma					
Mediastinal or hilar LNE	0.006	6.83	1.71–27.20	18.6% (11/59)	97.3% (177/182)
Adjacent organ invasion	0.004	4.14	1.58–10.82	33.9% (20/59)	90.7% (165/182)
Age (>60)	0.0001	3.88	1.94–7.78	55.9% (33/59)	70.9% (129/182)

Unless otherwise specified, for differentiating thymic bed cysts from thymomas.

† *P*‐values are from the multivariate logistic regression analysis for all variables

‡ “Water‐attenuated” indicates with a CT attenuation not bigger than 20 HU on either pre‐ or postcontrast scans.

CI, confidence interval; HU, Hounsfield unit; LNE, lymph node enlargement; OR, odds ratio.

*Thymic carcinoma versus thymoma*: In the univariate analysis, thymic carcinoma tended to be more ill‐defined (*P* < 0.0001), and to more frequently have focal cystic/necrotic changes (*P* = 0.002), adjacent organ invasion (*P* < 0.0001), pleural effusion (*P* = 0.004), and mediastinal LN enlargement (*P* < 0.0001). A significantly higher proportion of patients in their 60s or older were found in patients with thymic carcinoma (*P* = 0.0003).

In the multivariate analysis (Table 4), mediastinal LN enlargement (OR, 6.83; 95% CI, 1.71–27.20; *P* = 0.006), adjacent organ invasion (OR, 4.14; 95% CI, 1.58–10.82; *P* = 0.004), and age older than 60 years (OR, 3.88; 95% CI, 1.94–7.78; *P* = 0.0001) were significantly associated with thymic carcinoma. The corresponding sensitivity and specificity of the most strongly differentiating CT feature, mediastinal LN enlargement, were 18.6% and 97.3%, respectively. Representative cases are presented in Figure 5.

## Discussion

Our study reported detailed age‐ and gender‐specific distributions of resected malignant and benign anterior mediastinal lesions. The proportion of correct first‐choice MDCT diagnoses was still limited, ranging about 62.3% and gradually decreased as age increased regardless of genders from 75.4% in the 20s to 53.4% in ≥60s. This age‐dependent decrease of MDCT accuracy resulted from age‐dependent increase of thymic bed cyst and thymic carcinoma. The most strongly differentiating CT features for thymic bed cyst and thymic carcinoma were water attenuation and mediastinal LN enlargement, respectively. Those CT features had high specificities (98.4% and 97.3%, respectively), but low sensitivities (34.5% and 18.6%, respectively).

In accordance with a previous study reporting the age‐ and gender‐specific distribution of anterior mediastinal masses^3^ in patients <40 years, the prevalent diseases in our study were lymphoma, malignant germ cell tumor, and thymoma in men and lymphoma, thymoma, and benign teratoma in women. However, in patients in their 40s or older, thymoma, thymic bed cyst, and thymic carcinoma accounted for more than three‐fourths, unlike the previous study (thymoma and thyroid goiter). Patients with thyroid goiter were not included in this study, as it was typically diagnosed based on CT and rarely required pathologic confirmation. In addition, we separated thymic bed cyst and thymic carcinoma from thymoma.

There is a paucity of literature focused on the age‐specific proportion for thymic bed cyst and thymic carcinoma. While thymoma showed a constant proportion of around 30–40% regardless of age in patients ≥40 years, thymic bed cyst had an inverse V‐shaped relationship, with a peak in the 50s (42.1%), and thymic carcinoma had a gradual increase with age, with a peak in the 70+ group (30.0%). We confirmed the latter trend by comparing the mean ages of patients with thymoma and thymic carcinoma, as the mean age of patients with thymic carcinoma tended to be higher than those with thymoma, except for type A thymoma (mean age of thymoma, 63 years in type A; 54 years in type AB; 49 years in type B1; 48 years in type B2, 50 years in type B3; 57 years in type C).^10^

MDCT provided a proportion of correct first‐choice diagnoses of 62.3% (95% CI, 58.1–66.3%) for anterior mediastinal lesions which was very similar to that of single‐detector CT (61.4% [95% CI, 52.7–69.4%]).^2^ Likewise, our proportions of correct first‐choice MDCT diagnoses for thymic bed cyst (42.3%, [95% CI, 34.4–50.5%]) and for thymic carcinoma (30.5% [20.2–43.2]) were similar to the corresponding proportions on single‐detector CT (thymic cyst, 45.8% [22.3–71.4]; thymic carcinoma, 37.5% [16.4–64.7]).^2^

More than 80% of resected thymic bed cysts and thymic carcinoma were radiologically misdiagnosed as thymomas resulting in age‐dependent decrease of MDCT accuracy. Conventionally, CT differentiation of cysts from tumors is made based on the attenuation value of water (20 HU or less). However, only 34.5% of thymic bed cysts showed water attenuation in our study. Indeed, prior studies including a small number of thymic cysts reported that three‐fourths or more of thymic cysts had CT attenuation equal to that of the chest wall muscle, which was attributed to high calcium or protein contents, hemorrhage, or streak artifacts.^11^, ^12^ Focal necrosis existed in 33.3% of thymomas in our results, consistent with the results of other studies, where focal necrosis was found in 28–42% of thymomas. In accordance with previous studies,^2^, ^5^ thymic bed cysts tended to have a smooth contour and median location, while thymomas tended to be located off‐midline.

The differentiating CT features between thymoma and thymic carcinoma have been previously evaluated in a few studies.^13^, ^14^, ^15^ In our study, in the multivariate analysis, age older than 60, mediastinal or hilar LN enlargement, and adjacent organ invasion were found to be significant. Likewise, in the literature,^13^, ^14^, ^15^ mediastinal LN enlargement and adjacent organ invasion were characteristic CT features of thymic carcinoma, but were only present in 18.6% and 33.9% of cases, showing low sensitivity. Accordingly, even in cases suspected to be thymoma without those CT features, the possibility of thymic carcinoma should be considered, especially for patients older than 60.

Our study has some limitations. First, there was an inevitable selection bias due to its retrospective nature. Second, as we only included only surgically treated cases, the diagnostic accuracy of benign diseases, especially thymic bed cysts or thymic hyperplasia, might have been underestimated. Third, the CT scans were taken with various CT scanners, so the direct comparison of CT attenuation should be carefully interpreted. Finally, we only evaluated the role of conventional CT and did not explore the added role of other imaging techniques, such as dual‐energy CT, MRI, or PET.

In conclusion, according to the characteristic age‐specific distributions of malignant and benign anterior mediastinal lesions, especially for thymic carcinoma and thymic bed cysts, the MDCT accuracy decreased depending on age regardless of gender. The most strongly differentiating CT features (water attenuation for thymic bed cyst, mediastinal LN enlargement and adjacent organ invasion for thymic carcinoma) had high specificity but low sensitivity. Understanding this age‐dependent distribution and MDCT accuracy may potentially help make a correct diagnosis of anterior mediastinal lesions and considering additional noninvasive imaging work‐up including MRI.

## Disclosure

None of the authors has any potential conflicts of interest, including specific financial interests, relationships, or affiliations.

## Supporting information


**Table S1**. Age‐ and gender‐specific distribution of anterior mediastinal lesions.
