# Supplementary material for: Age‐ and gender‐specific disease distribution and the diagnostic accuracy of CT for resected anterior mediastinal lesions
Source: Thorac Cancer. 2019 Apr 29;10(6):1378–87. doi: 10.1111/1759-7714.13081 (PMC6558486; doi:10.1111/1759-7714.13081)
Supplement: Supplementary file 1 — Table S1. Age‐ and gender‐specific distribution of anterior mediastinal lesions. [file TCA-10-1378-s001.docx]

**Table S1: Age- and Gender-Specific Distribution of Anterior Mediastinal Lesions**

|  | **<30 M  (n=31)** | **<30 F (n=32)** | **30s M (n=34)** | **30s F (n=28)** | **40s M (n=56)** | **40s F (n=62)** | **50s M (n=62)** | **50s F (n=69)** | **60s M (n=66)** | **60s F (n=59)** | ≥**70 M (n=29)** | ≥**70 F (n=21)** |
| --- | --- | --- | --- | --- | --- | --- | --- | --- | --- | --- | --- | --- |
| **Malignancy** | **87.1%**  (70.5-95.5) | **90.6%**  (75.0-97.5) | **67.6%**  (50.8-81.0) | **53.6%**  (35.8-70.5) | **58.9%**  (45.9-70.9) | **66.1%**  (53.7-76.7) | **51.6%**  (39.5-63.6) | **53.6%**  (42.0-64.9) | **59.1%**  (47.0-70.1) | **66.1%**  (53.3-76.9) | **79.3%**  (61.3-90.5) | **66.7%**  (43.0-85.4) |
| **Lymphoma** |  |  |  |  |  |  |  |  |  |  |  |  |
| Aggressive lymphoma | 51.6%  (33.1-69.9) | 81.3%  (64.3-91.5) | 26.5%  (14.4-43.3) | 28.6%  (13.2-48.7) | 8.9%  (3.0-19.6) | 3.2%  (0.2-11.7) | 0.0%  (0.0-5.7) | 1.4%  (0.0-8.5) | 1.5%  (0.0-8.2) | 1.7%  (0.0-9.1) | 6.9%  (0.9-23.0) | 4.8%  (0.1-23.8) |
| Low-grade MALToma | 0.0%  (0.0-11.2) | 0.0%  (0.0-12.7) | 0.0%  (0.0-12.1) | 0.0%  (0.0-12.3) | 1.8%  (0.1-9.6) | 1.6%  (0.0-9.4) | 0.0%  (0.0-7.0) | 2.9%  (0.2-10.6) | 3.0%  (0.4-10.5) | 0.0%  (0.0-6.1) | 0.0%  (0.0-13.9) | 0.0%  (0.0-16.1) |
| **Germ Cell Tumor** |  |  |  |  |  |  |  |  |  |  |  |  |
| Malignant  germ cell tumor | 22.6%  (9.6-41.1) | 0.0%  (0.0-12.7) | 5.9%  (0.7-20.1) | 0.0%  (0.0-12.3) | 0.0%  (0.0-6.4) | 0.0%  (0.0-7.0) | 0.0%  (0.0-7.0) | 0.0%  (0.0-6.3) | 0.0%  (0.0-5.4) | 0.0%  (0.0-6.1) | 0.0%  (0.0-13.9) | 0.0%  (0.0-16.1) |
| **Thymic epithelial tumor** |  |  |  |  |  |  |  |  |  |  |  |  |
| Thymoma | 9.7%  (2.0-25.8) | 9.4%  (2.5-25.0) | 29.4%  (16.7-46.3) | 25.0%  (10.7-44.9) | 39.3%  (26.5-53.3) | 54.8%  (42.5-66.6) | 35.5%  (23.7-48.0) | 33.3%  (23.3-45.1) | 22.7%  (13.1-34.7) | 44.1%  (31.2-57.6) | 37.9%  (22.6-56.1) | 28.6%  (11.3-52.2) |
| Thymic carcinoma | 0.0%  (0.0-11.2) | 0.0%  (0.0-12.7) | 0.0%  (0.0-12.1) | 0.0%  (0.0-12.3) | 5.4%  (1.1-14.9) | 4.8%  (1.1-13.8) | 11.3%  (5.3-21.8) | 14.5%  (7.9-24.9) | 21.2%  (12.1-33.0) | 11.9%  (4.9-22.9) | 31.0%  (17.1-49.4) | 28.6%  (11.3-52.2) |
| **Lung cancer** | 0.0%  (0.0-11.2) | 0.0%  (0.0-12.7) | 0.0%  (0.0-12.1) | 0.0%  (0.0-12.3) | 0.0%  (0.0-6.4) | 0.0%  (0.0-7.0) | 3.2%  (0.2-11.7) | 0.0%  (0.0-6.3) | 6.1%  (1.7-14.8) | 3.4%  (0.4-11.7) | 3.4%  (0.0-18.6) | 4.8%  (0.1-23.8) |
| **Others (maignant)** | 3.2%  (0.0-16.7) | 0.0%  (0.0-12.7) | 5.9%  (0.7-20.1) | 0.0%  (0.0-12.3) | 3.6%  (0.4-12.3) | 1.6%  (0.0-9.4) | 1.6%  (0.0-9.4) | 1.4%  (0.0-8.5) | 4.5%  (1.0-12.7) | 5.1%  (0.3-19.0) | 0.0%  (0.0-11.6) | 0.0%  (0.0-16.1) |
| **Benign disease** | **12.9%**  (4.5-29.5) | **9.4%**  (2.5-25.0) | **32.4%**  (19.0-49.3) | **46.4%**  (29.5-64.2) | **41.1%**  (29.2-54.1) | **33.9%**  (23.3-46.3) | **48.4%**  (36.4-60.6) | **46.4%**  (35.1-58.0) | **40.9%**  (29.9-53.0) | **33.9%**  (23.1-46.7) | **20.7%**  (9.5-38.8) | **33.3%**  (14.6-57.0) |
| **Thymic bed cyst^a^** | 3.2%  (0.0-16.7) | 0.0%  (0.0-12.7) | 11.8%  (4.1-27.2) | 14.3%  (0.4-32.7) | 25.0%  (14.4-38.4) | 21.0%  (12.6-32.8) | 41.9%  (30.5-54.3) | 43.5%  (32.4-55.2) | 33.3%  (22.2-46.0) | 30.5%  (19.2-43.9) | 20.7%  (9.5-38.8) | 19.0%  (5.5-41.9) |
| **Thymic hyperplasia   or remnant** | 3.2%  (0.0-16.7) | 0.0%  (0.0-12.7) | 5.9%  (0.7-20.1) | 7.1%  (0.9-23.5) | 5.4%  (1.1-14.9) | 3.2%  (0.2-11.7) | 1.6%  (0.0-9.4) | 0.0%  (0.0-6.3) | 1.5%  (0.0-8.2) | 0.0%  (0.0-6.1) | 0.0%  (0.0-13.9) | 14.3%  (3.1-36.3) |
| **Benign teratoma** | 6.5%  (0.8-21.4) | 9.4%  (2.5-25.0) | 14.7%  (6.0-30.6) | 21.4%  (8.3-41.0) | 8.9%  (3.0-19.6) | 6.5%  (2.1-15.9) | 3.2%  (0.2-11.7) | 1.4%  (0.0-8.5) | 1.5%  (0.0-8.2) | 1.7%  (0.0-9.1) | 0.0%  (0.0-13.9) | 0.0%  (0.0-16.1) |
| **Others (benign)** | 0.0%  (0.0-11.2) | 0.0%  (0.0-12.7) | 0.0%  (0.0-12.1) | 3.6%  (0.1-18.4) | 1.8%  (0.0-9.6) | 3.2%  (0.2-11.7) | 1.6%  (0.0-9.4) | 1.4%  (0.0-8.5) | 4.5%  (1.0-12.7) | 1.7%  (0.0-9.1) | 0.0%  (0.0-13.9) | 0.0%  (0.0-16.1) |

Data is presented with percentage with its 95% confidence interval

^a^Thymic bed cysts include thymic cysts and bronchogenic cysts
